# Supplementary material for: Rapid Prototyping of Thermoplastic Microfluidic 3D Cell Culture Devices by Creating Regional Hydrophilicity Discrepancy
Source: Adv Sci (Weinh). 2023 Nov 30;11(7):2304332. doi: 10.1002/advs.202304332 (PMC10870023; doi:10.1002/advs.202304332)
Supplement: Supplementary file 1 — Supporting Information [file ADVS-11-2304332-s001.pdf]

ADVANCED SCIENCE
Open Access

Supporting Information

for *Adv. Sci.*, DOI 10.1002/advs.202304332

Rapid Prototyping of Thermoplastic Microfluidic 3D Cell Culture Devices by Creating Regional Hydrophilicity Discrepancy

Haiqing Bai, Kristen N. Peters Olson, Ming Pan, Thomas Marshall, Hardeep Singh, Jingzhe Ma, Paige Gilbride, Yu-Chieh Yuan, Jenna McCormack, Longlong Si, Sushila Maharjan, Di Huang, Xiaohua Qian, Carol Livermore, Yu Shrike Zhang and Xin Xie**

Supporting Information

Rapid Prototyping of Thermoplastic Microfluidic 3D Cell Culture Devices by Creating Regional Hydrophilicity Discrepancy

Haiqing Bai, Kristen N. Peters Olson, Ming Pan, Thomas Marshall, Hardeep Singh, Jingzhe Ma, Paige Gilbride, Yu-Chieh Yuan, Jenna McCormack, Longlong Si, Sushila Maharjan, Di Huang, Xiaohua Qian, Carol Livermore, Yu Shrike Zhang*, Xin Xie*

Dr. Haiqin Bai, Dr. Kristen N. Peters Olson, Dr. Ming Pan, Dr. Thomas Marshall, Dr. Hardeep Singh, Dr. Jingzhe Ma, Dr. Paige Gilbride, Dr. Yu-Chieh Yuan, Dr. Jenna McCormack, Dr. Xiaohua Qian, Dr. Xin Xie

Xellar Biosystems, Cambridge, MA, 02458, USA

Email: xxie@xellarbio.com (Xin Xie)

Prof. Longlong Si

CAS Key Laboratory of Quantitative Engineering Biology, Shenzhen Institute of Synthetic Biology, Shenzhen Institute of Advanced Technology, Chinese Academy of Sciences, Shenzhen 518055, P. R. China

Prof. Longlong Si

University of Chinese Academy of Sciences, Beijing 100049, P. R. China

Dr. Sushila Maharjan, Prof. Yu Shrike Zhang

[4]Division of Engineering in Medicine, Department of Medicine, Brigham and Women's Hospital, Harvard Medical School, Cambridge, MA 02142, USA

Email: yszhang@bwh.harvard.edu (Yu Shrike Zhang)

Prof. Di Huang

Research Center for Nano-biomaterials & Regenerative Medicine, College of Biomedical Engineering, Taiyuan University of Technology, Taiyuan 030024, P. R. China

Prof. Carol Livermore
Department of Mechanical and Industrial Engineering, Northeastern University, Boston, MA, 02115 USA

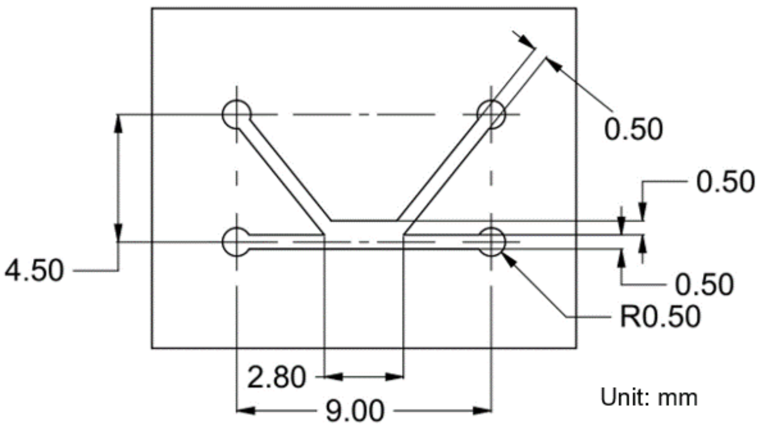

Figure S1. Schematic showing chip dimensions.

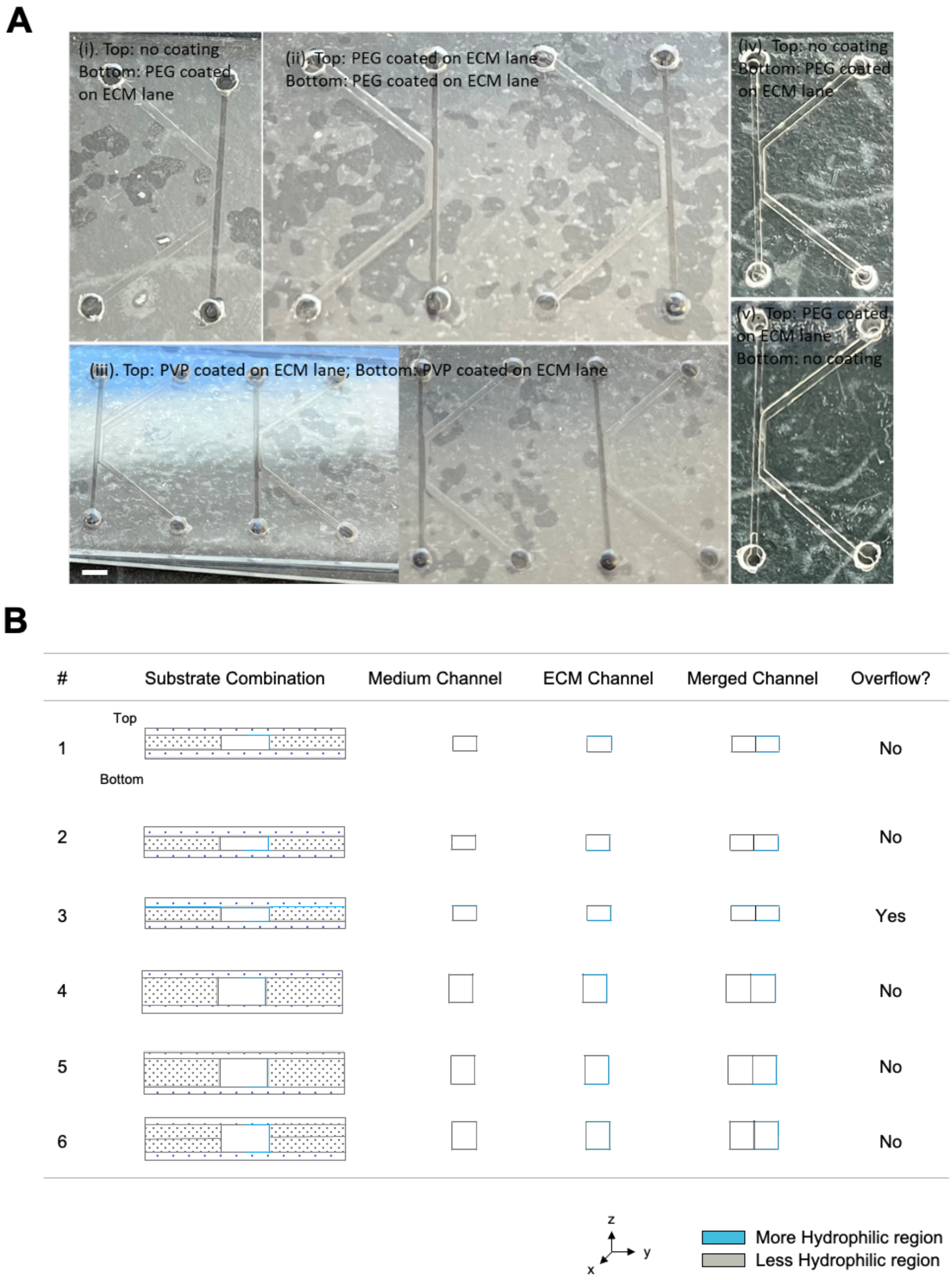

Figure S2. Effect of different coating configurations on hydrogel flow behavior. A) Photographs shows flow test results (before gelation) using chips (channel height: 120 μm) with different coating configurations using (i-iii). ~1.5 μL 0.35% rat collagen I solution; and (iv-v). ~1.5 μL 0.4% bovine collagen I solution, as hydrogel solution. Scale bar: 1 mm. **B**) Summary of hydrogel flow test using chips with different coating configurations.

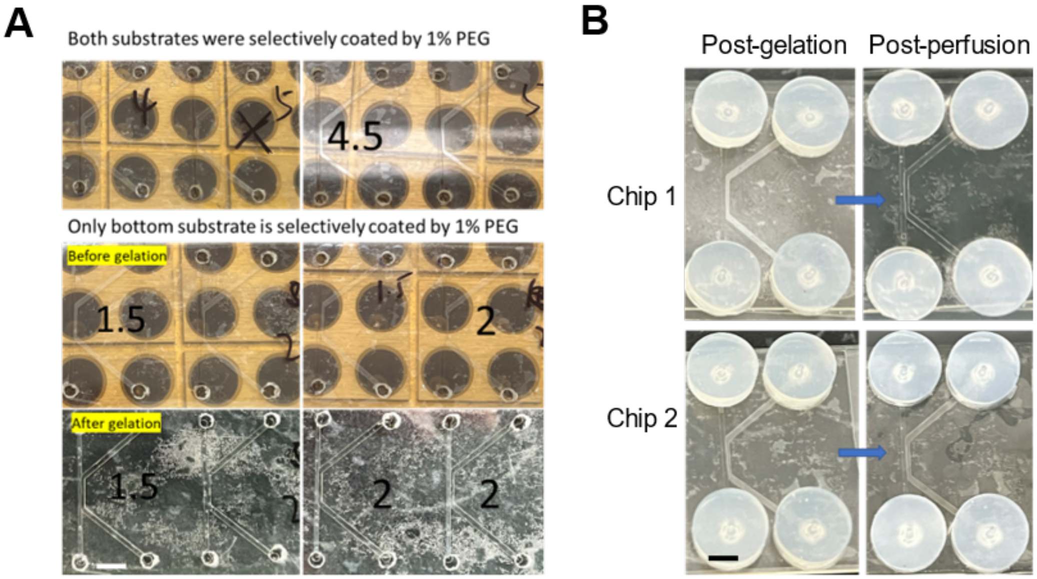

Figure S3. Effect of gamma irradiation on hydrogel flow behavior. A) Photographs showing results after loading 0.4% bovine collagen I solution into chips after sterilization by gamma irradiation at 25 kilogray (kGy). Numbers in the images are loading volumes. Scale bar: 2 mm. **B**) Photographs showing flowing and gelation results after loading ~1.5 µL 0.4% bovine collagen I solution into chips after sterilization by gamma irradiation at 25 kGy (channel height: 120 µm). The tubing connectors were adhered to the inlets and outlets between gel loading and gelation steps. After gelation the resulting chips are ready for perfusion experiments by flowing culture medium solutions across medium lane using a peristaltic pump. Scale bar: 2 mm.

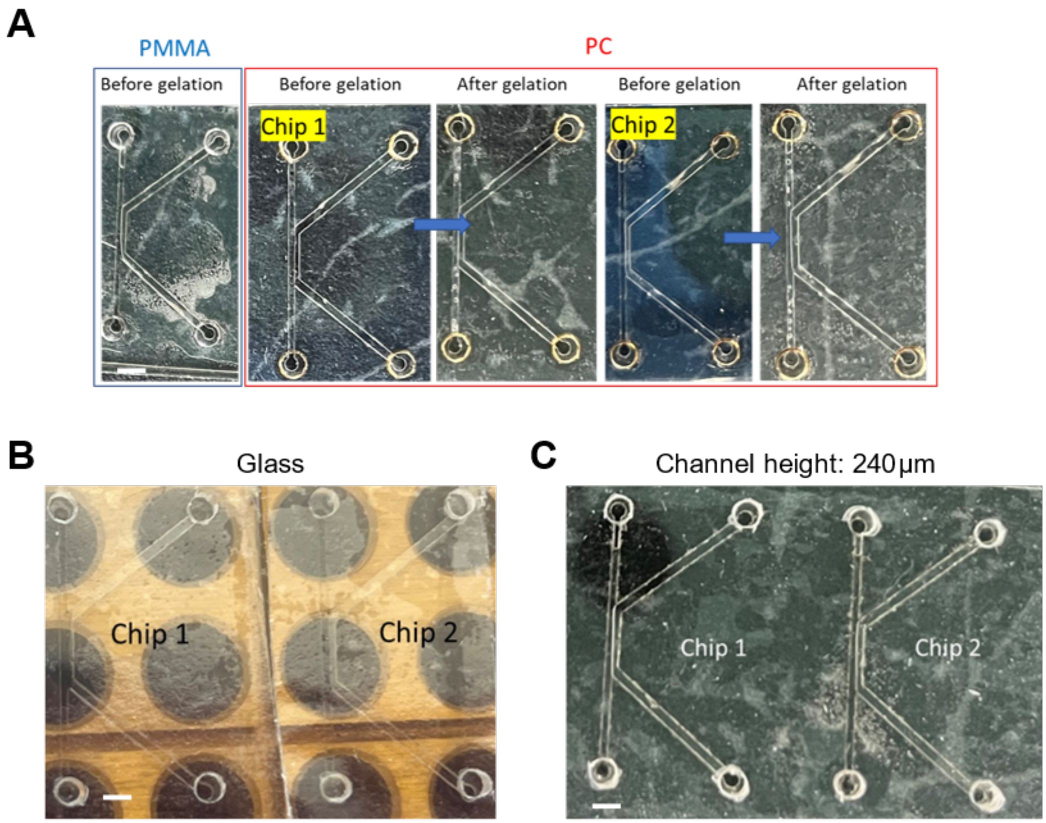

Figure S4. Effect of substrate materials and channel heights on hydrogel flow behavior. Photographs showing flowing and gelation results after loading 0.4% bovine collagen I solution into non-sterilized chips (channel height: 120 μm) with different types of plastic substrates. **A)** The top substrate was either PMMA or PC slide with ECM lanes coated by 1% PEG, the bottom substrate was pristine TC-PS slide without surface modifications. **B**) The top substrate was TC-PS slide with ECM lanes coated by 1% PEG, the bottom substrate was glass slide with ECM lanes coated by 1% PEG. **C**) The top substrate was TC-PS slide with ECM lanes coated by 1% PEG, the bottom substrate was TC-PS slide with ECM lanes coated by 1% PEG (channel height: 240 μm). Scale bar: 2 mm.

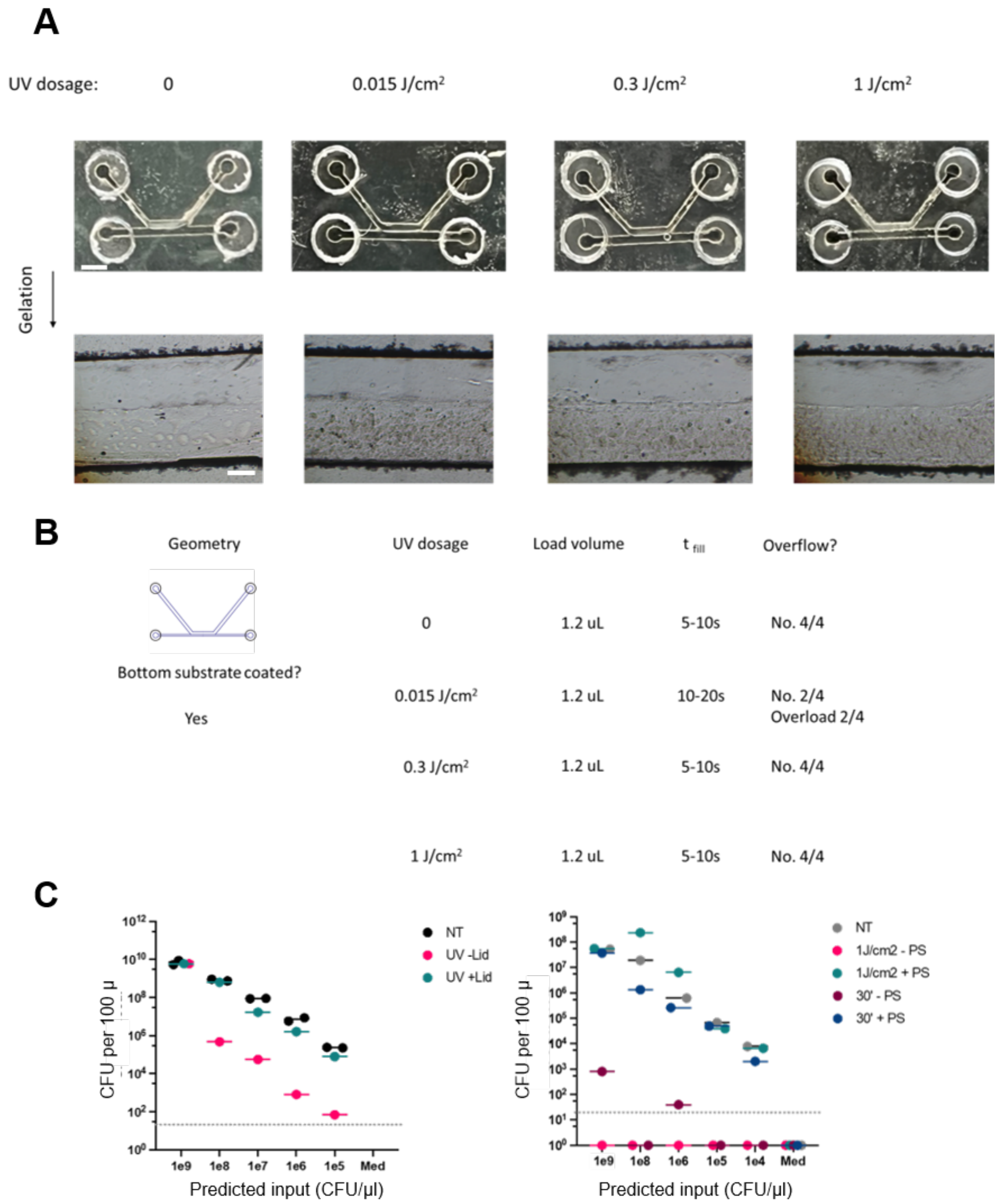

Figure S5. Effect of UV exposure on gel flow behavior and sterilization efficacy. A) Photographs showing the results of hydrogel flow tests where the ECM channel of the top substrate and the entire bottom substrate were coated by PVP. Both substrates were sterilized by UV at 254 nm with different energy levels. In all cases, the hydrogel solution only filled the ECM channel without significant overflow into the medium channel. Scale bars: 2 mm (top) and 250 µm (bottom). **B**) Summary on the effect of UV sterilization at different energy levels on hydrogel

solution flow behavior in Mercury chips. The entire bottom substrate was pristine without coating before UV sterilization. Note for results shown in the table, when hydrogel solution was overflowed, it filled the entire ECM and medium channel. **C**) Plots showing measured cell concentration from plate-well treated under various sterilization conditions, including different times or energy levels of UV exposure, whether the plate-well was covered PS film was covered during UV exposure.

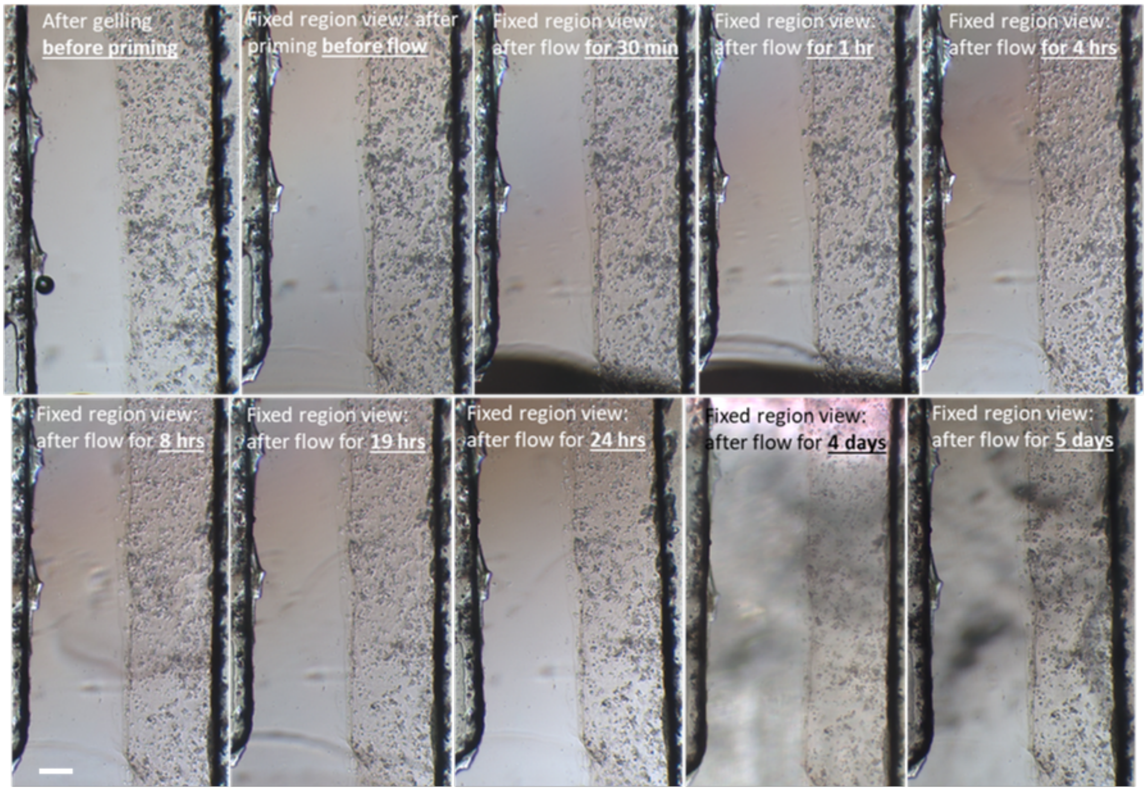

Figure S6. Gel Stability under perfusion. Optical microscope images showing gel and cell morphology under continuous flow of culture medium at 100 µl/hour. The chip was not sterilized, and the experiment was conducted on a microscope stage. The images were taken at different time points during flow test. Scale bar: 250 µm.

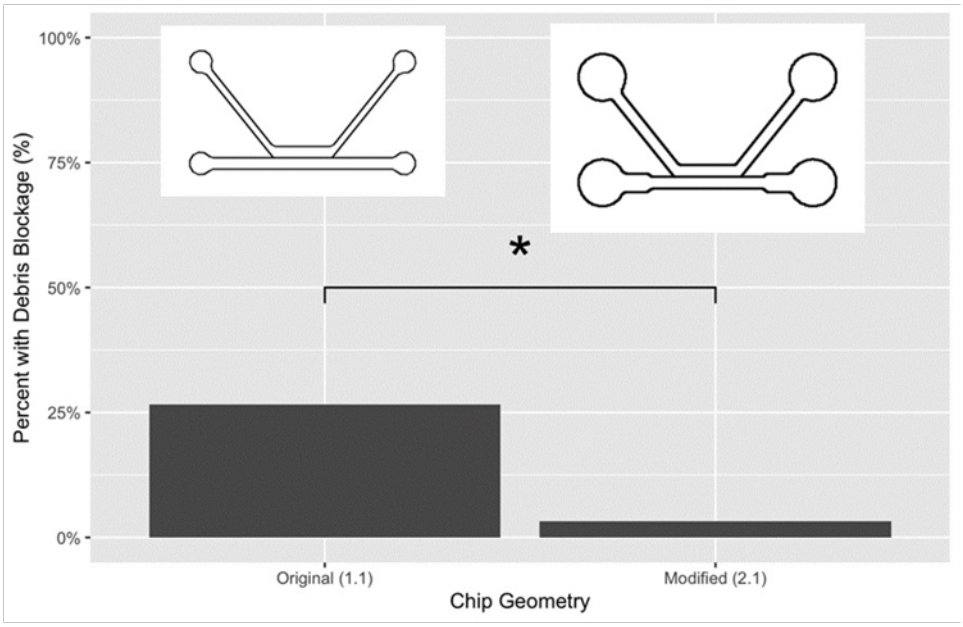

Figure S7. Effect of different channel geometries on the occurrence of channel blockage. Bar plot comparing the percentage of chips with debris between chip 1.1 and chip 2.1.

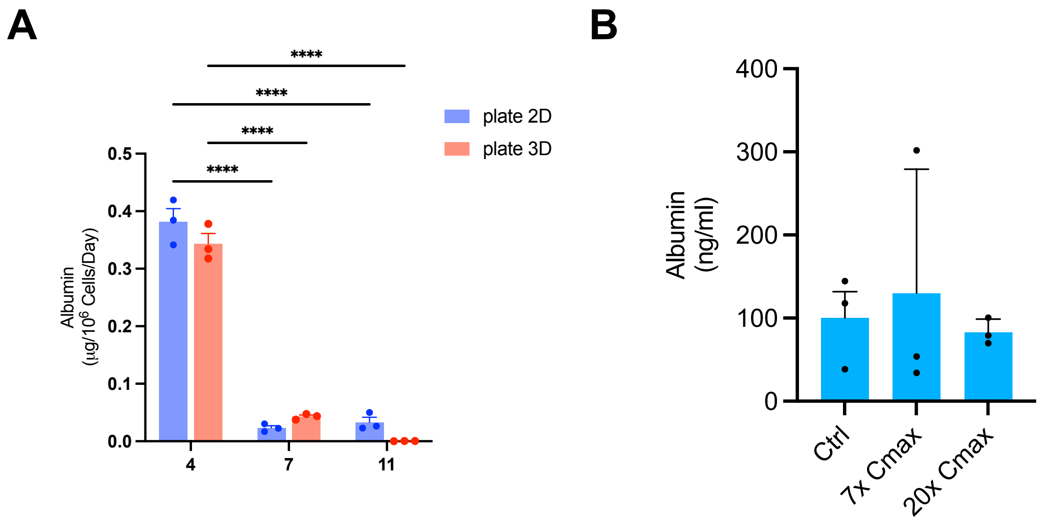

Figure S8. Comparing albumin production and troglitazone toxicity in 2D and 3D plate controls. A) Normalized albumin secretion levels in the culture medium at indicated days of culture in HepG2 2D and 3D plate controls. **B**) Albumin levels were measured from the HepG2 2D culture medium at 72 hours post- treatment at 7× Cmax and 20× Cmax concentration. Data represents mean ± SEM. N=3, one-way ANOVA with Tukey's post-hoc multiple comparisons correction, ****, p<0.0001.

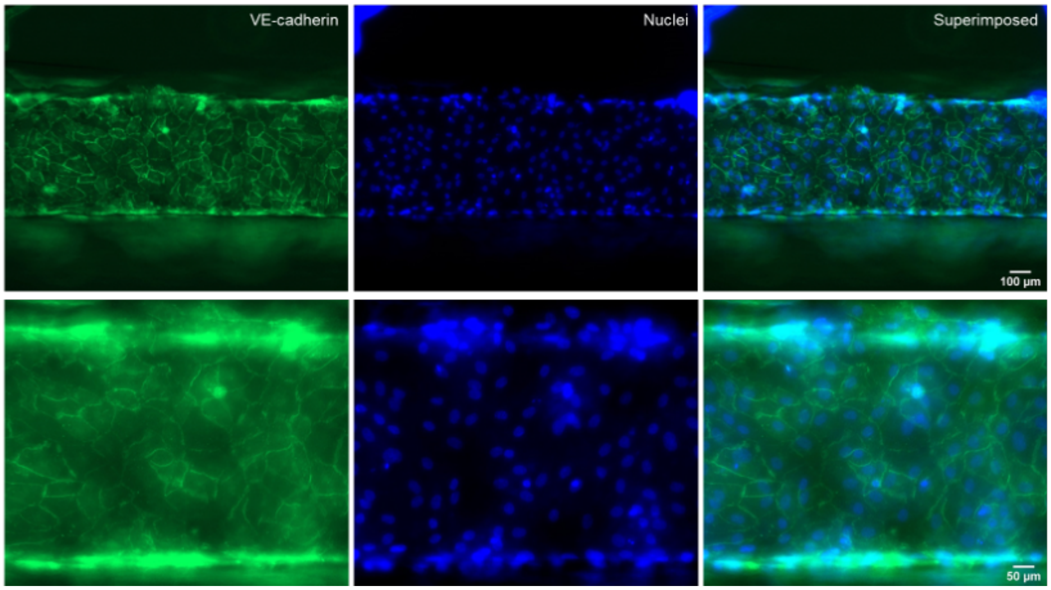

Figure S9. Feasibility of endothelial culture in the medium channel. Fluorescence micrographs showing HUVECs stained for VE-cadherin (green) and nuclei (blue), in the channel at day 3 of cell culture.

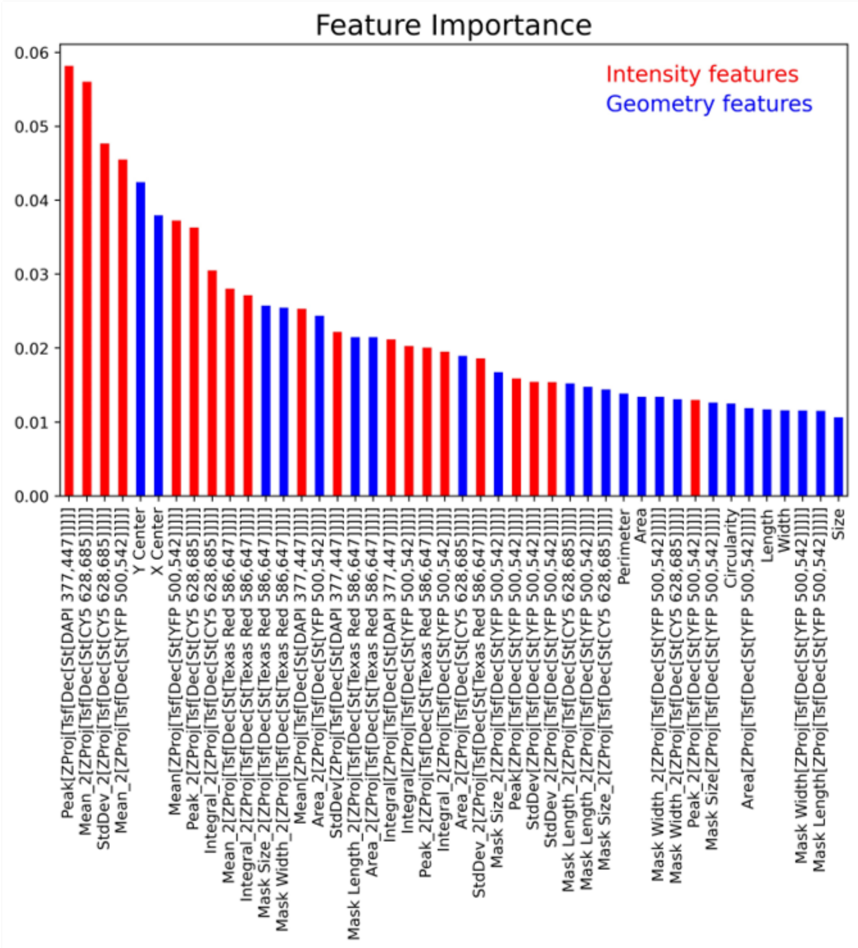

Figure S10. Morphological analysis of CellPainting images. Bar graph showing feature importance ranking color-coded by either intensity-based features or geometry features. Note that the top-ranking features are intensity features.

Table S1. Staining reagents and imaging settings for CellPainting.

Staining Reagents	Cellular Compartment	Vendor Cat. No.	Imaging Channel	Ex (nm)	Em(nm)
MitoTracker Deep Red	Mitochondria	Invitrogen #M22426	Cy5	623	628/685
Phalloidin/ Alexa Fluor 568	Cell Membrane	Invitrogen #A12380	TxRed	590	586/647
Wheat-Germ Agglutinin/ Alexa Fluor 555	F-actin Cytoskeleton	Invitrogen, #W32464	TRITC	554	556/600
SYTO14 Green Fluorescent Nucleic Acid Dye	Nucleoli, Cytoplasmic RNA	Invitrogen, #S7576	YFP	505	500/542
Concanavalin A/ Alexa Fluor 488	Endoplasmic Reticulum	Invitrogen #C11252	GFP	465	469/525
Hoechst 33342	Nucleus-dsDNA Selective	Thermo Fisher Scientific #62249	DAPI	365	377/447

Table S2. Conditions for staining reagents.

Staining Solution Type	Component Dye and Concentration	Solvent
**Live-Cell**	MitoTracker Deep Red (500 nM)<br>Wheat-Germ Agglutinin/Alexa Fluor 555 (1.5 μg/mL)	1×PBS
**Fixed-Cell**	Phalloidin Alexa Fluor 568 (165 nM)<br>SYTO14 Green Fluorescent Nucleic Acid Dye (3 μM)<br>Concanavalin A/Alexa Fluor 488 (50 μg/mL)<br>Hoechst 33342 (20 μg/mL)	1×PBS
